# Abstract withdrawn

**DOI:** 10.1186/cc11712

**Published:** 2012-11-14

## Abstract withdrawn

